# Land Cover Classification Based on Airborne Lidar Point Cloud with Possibility Method and Multi-Classifier

**DOI:** 10.3390/s23218841

**Published:** 2023-10-31

**Authors:** Danjing Zhao, Linna Ji, Fengbao Yang

**Affiliations:** School of Information and Communication Engineering, North University of China, Taiyuan 030051, China

**Keywords:** possibility theory, classifier fusion, land cover classification, point cloud, SVM, ALS

## Abstract

As important geospatial data, point cloud collected from an aerial laser scanner (ALS) provides three-dimensional (3D) information for the study of the distribution of typical urban land cover, which is critical in the construction of a “digital city”. However, existing point cloud classification methods usually use a single machine learning classifier that experiences uncertainty in making decisions for fuzzy samples in confusing areas. This limits the improvement of classification accuracy. To take full advantage of different classifiers and reduce uncertainty, we propose a classification method based on possibility theory and multi-classifier fusion. Firstly, the feature importance measure was performed by the XGBoost algorithm to construct a feature space, and two commonly used support vector machines (SVMs) were the chosen base classifiers. Then, classification results from the two base classifiers were quantitatively evaluated to define the confusing areas in classification. Finally, the confidence degree of each classifier for different categories was calculated by the confusion matrix and normalized to obtain the weights. Then, we synthesize different classifiers based on possibility theory to achieve more accurate classification in the confusion areas. DALES datasets were utilized to assess the proposed method. The results reveal that the proposed method can significantly improve classification accuracy in confusing areas.

## 1. Introduction

Land cover classification is a focus of research among photogrammetry and remote sensing communities [1]. With the booming development of urban areas and the urbanization shift of population, the demand for urban land cover classification is dramatically increasing [2,3]. Urban areas usually consist of a complexity combination of natural and artificial surfaces, which makes urban land cover classification challenging. As a popular mean of active remote sensing (RS), light detection and ranging (LiDAR) can acquire 3D point clouds and also allow rapid access to information over large areas, which is widely used in urban land cover classification [4,5]. Accurate urban land cover classification is crucial and necessary for many applications, such as environmental monitoring [6], urban planning [7], and resource management [8].

Machine learning algorithms were widely used in automated land cover classifications over the past decade [9,10], with reasonable classification accuracy in remote sensing applications [11,12,13]. Machine learning techniques were extensively employed in land use/land cover change (LUCC) studies using remotely sensed data delivered by spaceborne platforms [14,15,16,17,18]. For example, Lin et al. [19] integrated Sentinel-2 multispectral surface reflectance and vegetation indices, and lidar-based canopy height and slope to generate a RF model for three-level LULC classification. Din et al. [20] used the Gaussian-based radial basis function (RBF) kernel for training, within a SVM-supervised classification framework to retrieve LULC maps from Landsat datasets. Classification and regression trees (CART) were also used to perform the classification [21]. Although these methods achieve better classification, they are restricted to two-dimensional information obtained through remotely sensed images. In contrast, point cloud data contains three-dimensional coordinates of objects along with attributes such as reflection intensity and color, which can describe and distinguish different categories more accurately. For example, Liao et al. [22] integrate point cloud supervoxels and their locally convex-connected patches into a random forest (RF) classifier. Chen et al. [23] proposed a new point cloud classification algorithm of the mixed kernel function support vector machine (SVM) to distinguish different types of ground objects. Many other scholars compared these algorithms; for example, Huang et al. [24] classified the land cover in Ho Chi Minh City by comparing three classification algorithms, i.e., the back propagation neural network, SVM, and RF. However, the performance of a single method in confusing areas, such as boundaries and covered by thin point clouds, is often degraded due to the potential interclass similarity and intraclass inconsistency of objects, which prompted researchers to explore more accurate point cloud classification models [25]. Regarding misclassification due to interclass similarity and intraclass inconsistency, investigating uncertainty issues in the classification process is crucial to ensuring the accuracy of land cover classification [26]. The risk exists when two categories are indistinguishable at the category boundary. The characteristics of the two categories are similar, which is mainly about the “different data features for the same class” and “same data features for different classes”. Moreover, under certain circumstances, a single classifier may make it difficult to solve the problem of classification uncertainty in confusing regions [27]. Multiple classifier systems (MCSs) outperform a single classifier based on an assumption that a set of diverse classifiers causes individual errors, which are unlikely to be produced by the majority of other classifiers. Multi-classifier fusion algorithms [28] can effectively solve the uncertainty in classification [29].

In multi-classifier fusion, base classifiers refer to individual classifiers used in classification. The problem that a single base classifier cannot achieve can be effectively solved by fusion of these base classifiers in a MCS [30,31]. Using multiple classifiers and fusing the results with Dempster–Shafer (DS) evidence theory [32,33] can significantly enhance classification performance. However, existing methods tend to ignore significant variability among member classifiers in an integrated system. Therefore, variability and correlation between the performance of member classifiers need to be further explored for quantifying their contributions to the integrated classifier, which can lead to greater classification results. The possibility theory provides a new approach to address uncertainty problems [34]. Possibility synthesis is the process of combining different information sources according to certain rules to obtain a more accurate and reliable description, and it is widely used in image fusion [35] and risk assessment [36]. In multi-classifier fusion, possibility synthesis can measure the differences based on the characteristics of data, study the synergistic method of multiple classifiers, and utilize the information obtained to create a comprehensive description of a complex system to improve its effectiveness [37]. The synthesis of the base classifiers using possibility theory can effectively measure the differences between the classifiers and improve the reliability of the classification results.

In this paper, we present an effective approach for urban land cover classification from LiDAR point clouds and co-registered visible images based on SVM and possibility theory. It employs SVM classifiers in the initial classification and then adapts the possibility theory for the post-processing. Firstly, we define confusing areas through the classification uncertainty quantitatively, then, an optimized possibility theory is applied for multi-classifier fusion to improve the classification accuracy. However, algorithmic complexity and computational power must be considered when performing large-scale fusion computations. Therefore, by synthesizing samples with high uncertainty regions, we reduce the number of computations in this paper. Meanwhile, we choose simple weighted synthesis operators to reduce the complexity. The main contributions of our work can be summarized as follows:

1. A method for defining confusion areas is proposed to classify the pre-classification results into uncertainty regions. These regions will be used as an important basis for processing uncertainty information in later studies.
2. In the optimization process of the confusing area, the possibility theory fully considers the classification advantages of the base classifiers for different classes and the correlation between the two base classifiers. It can effectively reduce the influence of conflicts on the fusion results and achieve more accurate results.

The rest of the paper is organized as follows: In Section 2, related work is presented. The methodology for novel land cover classification is presented in Section 3. The experimental results and discussions are presented in Section 4. Section 5 concludes the work.

## 2. Literature Review

The literature review covers both traditional and deep learning-based point cloud classification methods.

Traditional point cloud classification: Traditional point cloud classification methods are mainly based on manually extracting feature descriptors from the point cloud as the input to classifiers [38,39,40]. These methods can be divided into unsupervised and supervised [41,42]. The unsupervised classification method is used in the classification of RS fields [43,44]; for example, k-means clustering, nearest-neighbor mapping, and iterative self-organizing data (ISODATA) [45]. However, without comprehensive analysis and clear guidance on initial parameters before training, reliable classification results may not be obtained, leading to inconsistency with the true class. On the other hand, although supervised classification algorithms require labeled training samples, they usually have better classification accuracy compared to unsupervised classification methods [40,46,47]. Popular supervised learning methods include SVM, RF, artificial neural network (ANN), CART, maximum likelihood classifier (MLC), and extreme gradient boosting (XGBoost). Among these traditional classification algorithms, SVM is a prominent method that is successful in several applications due to its high accuracy and robustness [28,48].

Deep learning-based point cloud classification: Deep learning showed excellent performance in many computer vision tasks, such as convolutional neural networks (CNN), which became the algorithms for classification, segmentation, and detection [49,50,51]. It was also explored in 3D point cloud classification research [52,53,54,55,56]. CNNs are initially applied to data with structured grids, such as images. The unstructured nature of point clouds makes it difficult to directly apply CNNs in 3D point cloud classification [57,58]. Previous approaches to preprocessing point clouds into a structured grid format can be broadly classified into two categories: voxel-based and multiview-based [59]. Voxel-based methods convert point clouds into a 3D voxel structure of size *X* × *Y* × *Z* and convolve it with 3D kernels. However, these methods suffer from high memory consumption due to density and complexity of the original point cloud, which generates a large number of sparse voxels. Multi-view methods project point cloud data from different directions onto a two-dimensional plane and use these 2D views as input to a 2D CNN model. This approach provides an effective way to leverage the strengths of 2D CNNs and extend them to handle three-dimensional point clouds [60,61]. Recently, state-of-the-art approaches to deep learning techniques that can operate directly on point clouds are emerging [62,63]. Qi et al. [64] proposed PointNet to apply deep learning models directly on the raw point cloud; the model is unable to obtain complete local feature information, and since then, many improved networks emerged [65,66], including PointCNN [67], PointSift [68], D-FCN [69], PointLK [70], KPConv [71], PV-RCNN [72], and so on. Due to the complex multi-layer structure, deep learning models require a large amount of labeled training data and computational power as compared to traditional machine learning methods [73]. In cases where it is difficult to have a large amount of labeled training samples, traditional machine learning shows its advantages with lower computational cost and higher interpretability compared to deep learning models.

In this paper, we adapt multiple SVMs with possibility theory in fusion to explore an effective approach for urban land cover classification from LiDAR point clouds. The focus of this study is to tackle misclassification in confusing areas, such as boundary areas. 

## 3. Methods

**Figure 1:**
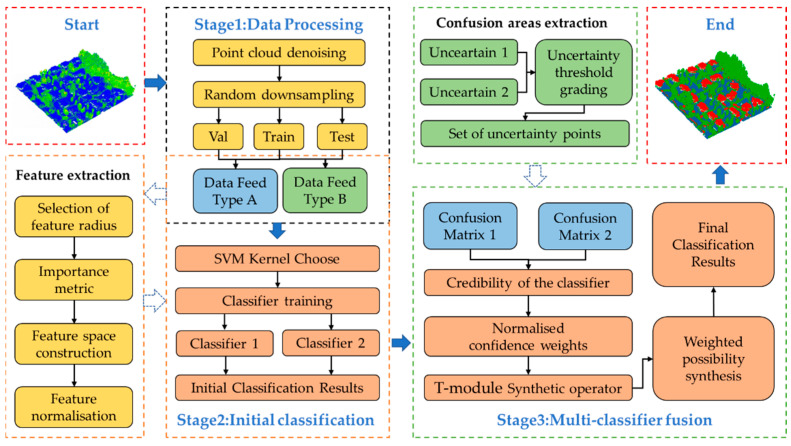
Workflow of the proposed method.

The research was established on possibility theory to fuse results from muti-classifiers. As shown in Figure 1, the proposed approach consists of three stages, i.e., data processing, initial classification, and multi-classifier fusion. In the stage of data preprocess, considering that a different data type as an input can provide a reference for the definition of confusing regions and the selection of synthetic weights needed in our method, we define two data input methods in the data processing section, in which the synthetic weights can be obtained according to the results when we input the training and validation datasets into the initial classifier, and the confusing areas are defined according to the results when we input the training and test dataset into the initial classifier. Secondly, features of the point cloud are extracted and selected using the XGBoost algorithm [74] to construct the feature space by calculating the local geometric features of the point cloud local features with different scale radii in the range of 0.5–2.5, where “scale radius” is the size of the neighborhood. Then, four kernel functions are used to train the SVM classifiers separately to obtain the pre-classification results, followed by selecting base classifiers and identifying the confusing areas by setting grading thresholds, which can divide the classification results into regions of high uncertainty, low uncertainty, and lower uncertainty. Finally, the classification confidence degree of different categories is obtained based on the confusion matrix and the weights are calculated, and the classification results of confusing areas are then synthesized based on the T-module operator to obtain the class judgment. 

### 3.1. Study Data and Area

The airborne LiDAR data used in the study are the DALES dataset published by the University of Dayton, which contains over half a billion hand-labeled points covering 10 km^2^ areas and eight object categories. The data were collected using a Riegl Q1560 dual-channel system (Riegl Laser Measurement Systems GmbH, City of Vienna, Austria) flown in a Piper PA31 Panther Navajo (Piper Aircraft, Inc., Vero Beach, Florida) [75]. The total aerial LiDAR collection covered 330 km^2^ over the city of Surrey in British Columbia, Canada. In our study, an area is selected from the DALES dataset to verify the effectiveness of the multi-classifier fusion method. The density of the original point cloud dataset is 50 points per square meter.

**Figure 2:**
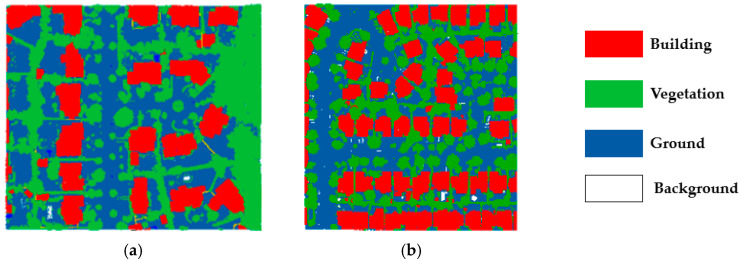
Experimental area: (**a**) Dataset 1; (**b**) Dataset 2.

To obtain the experimental data, data processing is conducted in the first stage, in which the original point cloud is denoised and randomly downsampled. The denoising is carried out using a statistical outlier removal to remove sporadic points. This filter only removes an average of 11 points per tile, but drastically reduces the overall bounding box in the Z direction, resulting in a 50% reduction. The average density of point cloud samples after pre-processing was 15 points per square meter. Then, the obtained 800,000-points data were divided into training, validation, and testing datasets in a ratio of 6:2:2. The whole scene was divided into four types: buildings, vegetation, ground, and background. The experimental area is shown in Figure 2, where (a) is the Dataset 1 and (b) is the Dataset 2.

### 3.2. Feature Space

Considering that some features are extracted from the original point cloud (before down sampling) with better robustness, we propose two new types of features, i.e., single-point features and local features from the original point cloud. Local features are extracted from a set of neighboring points. By selecting a local neighborhood of each point in the point cloud, features can be computed based on the spatial arrangement of 3D points in the neighborhood. Among the listed five features below, (1) and (3) are local features, and (2), (4), and (5) belong to the single point features. In (1), there are five different features to represent the local 3D shape.

(1) Local 3D shape features: Covariance features can characterize the 3D spatial distribution of local points. For a given 3D point X and its neighbors, the corresponding derived three normalized eigenvalues $\lambda_{1}$, $\lambda_{2}$, and $\lambda_{3}$ can be obtained, which can be used to calculate a set of local 3D shape features, including ominvariance $O_{\lambda }$, curvature $C_{\lambda }$, linearity $L_{\lambda }$, sphericity $S_{\lambda }$, eigenentropy $E_{\lambda }$, surface variation, and verticality. The definitions of these features are shown in Equations (1)–(5).
  (1) $$O_{\lambda }=\sqrt[{3}]{\lambda_{1}\lambda_{2}\lambda_{3}}$$
  (2) $$C_{\lambda }=\frac{\lambda_{3}}{\lambda_{1}+\lambda_{2}+\lambda_{3}}$$
  (3) $$L_{\lambda }=\frac{\lambda_{1}-\lambda_{2}}{\lambda_{1}}$$
  (4) $$S_{\lambda }=\frac{\lambda_{3}}{\lambda_{1}}$$
  (5) $$E_{\lambda }=\sum_{i=1}^{3}\lambda_{i}\ln\lambda_{i}.$$

(2) Number of neighbors: A chosen number of neighborhood points can provide local contextual information without introducing excessive noise. This helps the feature extraction model to better understand the local structure and shape features of the point cloud and achieve more accurate classification results.
(3) Roughness: it can characterize the distance between the point cloud and the best-fit surface calculated from the nearest neighbor, which reflects the undulation and erosion of the ground surface and different feature types.
(4) Height: it refers to the effect of ground undulation on elevation features, which can better distinguish between land cover with widely varying elevations.
(5) LiDAR echoes: A transmitted laser pulse is returned to the LiDAR sensor as single echoes (Ns) and multiple echoes (Nm). For impenetrable ground, there is only one reflected echo, while the laser spots can penetrate vegetation and therefore provide multiple echoes.

A spherical neighborhood refers to the points contained within a sphere with a point as the center and r as the radius. In this study, we select the closest neighbors based on 3D distances and a spherical neighborhood with a flexible radius. The neighborhood characteristics of the point cloud are calculated and visualized by setting the range of R neighborhood values in such a way that the optimal neighborhood radius can be selected. If there are not enough neighbors to compute a quadric (i.e., less than 6), an invalid scalar value (NaN) is set for the point. Therefore, the radius of the neighborhood should be chosen so that there are as few invalid points as possible.

Selecting a set of appropriate features can not only avoid the loss of computing efficiency caused by feature redundancy, but also ensures classification accuracy. The XGBoost algorithm is used to measure the importance of each feature [64]. XGBoost intelligently identifies the importance scores of features through the construction of boosted trees, and the features that are used most in boosting the trees make key decisions and have the highest scores. To calculate the importance of a single decision tree is to weigh the relative value of a feature observation and the number of times the feature is used to split the data across all trees. The feature importance of all decision trees is then averaged to give the resulting value, i.e., the more frequently an attribute is used to build a decision tree in the model, the higher is its relative importance. The feature importance is expressed as Equation (6).

(6) $$importance=\frac{score}{\sum_{i=1}^{N}score_{i}}$$

where N is the number of features, and score is an output from the XGBoost via the boosted trees algorithm.

In this paper, the features are ranked based on their importance. Firstly, all features are used as an input to the classifier. The feature selection is conducted by iteratively removing the feature ranked lowest in the input until the classification accuracy starts dropping. The selected features are normalized to prevent a feature from having too much influence. The processed data have a mean of 0 and a standard deviation of 1, which satisfies the standard normal distribution. The normalization is as shown in Equations (7) and (8).

(7) $$y^{\prime }=(y-\mu )/\sigma$$

(8) $$\sigma =\sqrt{\frac{\sum_{i=1}^{n}{\left(x_{i}-mean\right)}^{2}}{n}}$$

where μ is the sample mean and σ is the sample standard deviation. In Equation (8), $x_{i}$ is the original data, n is the total number of data, and mean represents the average value.

### 3.3. Initial Classification by SVM Classifiers

In the second stage of our method, the initial classification is performed by SVMs with different kernel functions. To obtain results at the initial classification stage, four kernel functions are used to train the SVM classifiers separately, and two with higher pre-classification results are selected as the base classifiers. The different results obtained based on different input data types can be used as an essential contribution to the multi-classifier synthesis in our methodology, a classification model optimized globally that finds the best hyperplane to linearly separate data with different classes. However, most data are not linearly separable. Therefore, SVM uses kernel functions to map the multidimensional data into a high-dimensional space to increase the separability. Different kernel functions can be chosen in different cases, and commonly used kernel functions are polynomial kernel function (Poly), radial basis function (RBF), and S-shaped kernel (Sigmoid), are shown in Equations (9)–(11), respectively.

(9) $$k(x_{1},x_{2})={\left(x_{1}x_{2}+c\right)}^{d}$$

where d represents the polynomial degree, and c is a fixed parameter.

(10) $$k(x_{1},x_{2})=\exp(-\gamma *\left\|x_{1}\right.-{\left.x_{2}\right\|}^{2}/2\sigma^{2})$$

where σ is a parameter depended on gamma (γ) parameter, which controls the width of the kernel.

(11) $$k(x_{1},x_{2})=\tanh(\gamma x_{1}{}^{T}x_{2}+\theta )$$

where γ is the parameter that controls the shape of the kernel function curve and θ is a fixed parameter.

**Figure 3:**
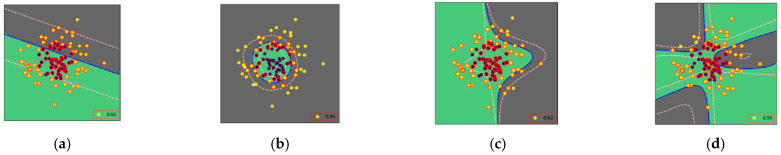
Comparison of SVM kernel function: (**a**) SVM-Linear; (**b**) SVM-RBF; (**c**) SVM-Poly; and (**d**) SVM-Sigmoid.

Different kernel functions are used in SVMs to classify LiDAR point clouds represented by a feature vector and then to calculate the classification accuracy. The spatial distribution of the point cloud is not regular and there is no topological relationship between the points; therefore, it is scattered. Figure 3a–d demonstrates a simulation of SVM in classification using a linear, polynomial, radial basis, and S-shaped functions, respectively, where yellow and blue points represent two different classes. The aim of the SVMs is to fit curves that can distinguish between the two classes. By evaluating the curves, the SVM classifiers can achieve their potential performance with less misclassification (indicated by red in Figure 3).

Figure 3 shows that it is not always easy to find a hyperplane that can separate the data by a kernel function mapping, as some boundary points and misclassified points are there, as shown in the red point area.

### 3.4. Definition of Confusing Areas

In LiDAR data, there must be problems such as “different data features representing the same class” and “same data features for different classes”. From the point of view of uncertainty information processing, the inaccurate classification results are due to the fact that the features cannot point to a particular category, i.e., the attribute boundaries between different classes are not obvious. Therefore, from the initial classification results of the base classifiers, we set the hierarchical threshold of the prediction probability value (predict_proba) of each class by the percentile truncation method to determine confusing areas. For example, in a two-classification problem, the preliminary classification results of the data to be classified are 0.51 and 0.49, respectively, which means that the probability of the data being category A is 0.51, and the probability of the data being category B is 0.49. According to the principle of maximum probability, the data should be classified as category A. However, the gap between the two categories is very similar, and the category boundaries cannot be divided. Our approach therefore focuses on solving such problems. The point cloud with a prediction probability of [60%, 65%) and [40%, 45%) is a region with low uncertainty, [45%, 60%) is a region with high uncertainty, and the rest are regions with lower uncertainty. The high uncertainty areas in the two classifiers are merged as confusing areas.

### 3.5. Improvement of Possibility Theory in Fusion of Results from Multi-Classifiers

We adapt possibility theory to solve the misclassification problems in the defined confusing areas. Possibility theory provides an effective way for processing the uncertainty problem, and it was first introduced in 1978 by Zadeh [76], who gave a fuzzy set pre-planarization of possibility theory. The commonly used synthesis method uses some operators, which mainly include the T-module operator, S-module operator, and mean operator. The T-module operator is suitable for the case where there is a large overlap and it can effectively handle the redundancy of information. In addition, the T-module operator considers the correlation between different data sources, thus showing different forms of synthesis that can provide us with more choices. The common manifestations of T-module operators are explained as follows, where x and y are two data sources, i.e., two classifiers, and q represents the operator span.

When x and y are locally positively correlated (q=1), the T-module operator is expressed as in Equation (12).

(12) $$T(x,y)={\left(x^{-1}+y^{-1}-1\right)}^{-1}$$

when x and y are lightly positively correlated (q=0.5), the T-module operator is expressed as in Equation (13).

(13) $$T(x,y)={\left(x^{-0.5}+y^{-0.5}-1\right)}^{-0.5}$$

when x and y are not related (q→0), the T-module operator is expressed as in Equation (14).

(14) T(x,y)=x⋅y

when x and y have extremely negative correlation (q=−1), the T-module operator is expressed as shown in the following Equation (15):

(15) T(x,y)=max(0,x+y−1).

T-module operator synthesis is selected for the two classifiers. Correlation coefficient is a statistical indicator of the strength of the relationship between two variables. The range of the coefficient is between −1 and +1. It can generally be classified into three levels: |r| < 0.4 for light correlation; 0.4 ≤ |r| < 0.7 for local correlation; and 0.7 ≤ |r| < 1 for extreme correlation. We define a negative correlation when r < 0 and a positive correlation when r > 0.

The fusion rule used in conventional possibility theory treats all evidence, in this case classifiers, with equal importance. This disregards the varying importance of different evidence. In this study, taking the classification accuracy of base classifiers in the fusion rule, we propose a multi-classifier fusion method based on a weighted T-module operator to synthesize the SVM classification results from different base classifiers. Different classifiers have different abilities to identify the same class. If the uncertainty of the classification results is high, the classifier will contribute less to the true classes. Therefore, smaller weights should be assigned. 

Suppose there is a classification task to identify k-classes in the dataset X, and there are N samples in total. A confusion matrix $R_{k}(k=1,2)$ showing classification results from the data pre-classification is expressed in Equation (16).

(16) $$R_{k}=\left[\begin{array}{ccc} R_{k11} & R_{k12} & R_{k13}\\ R_{k21} & R_{k22} & R_{k23}\\ R_{k31} & R_{k32} & R_{k33} \end{array}\right]$$

where the diagonal elements denote the number of categories correctly classified by the classifier, and the non-diagonal elements denote the number of categories incorrectly classified by the classifier. Accordingly, the classification accuracy of the classifier k is calculated for different categories, as in Equation (17).

(17) $$C_{kij}=\frac{R_{kij}}{\sum_{j=1}^{3}R_{kij}}$$

where $R_{kij}(i=1,2,3;j=1,2,3)$ is the percentage of the total number of samples of class i judged by the classifier to be class i. The credibility of class classification represents the support process when judging the target type. When the classifier k outputs a class i, it is the probability that the true class of the current sample is i. We define the classification credibility as shown in Equation (18).

(18) $$C_{ki}=\frac{C_{kii}}{\sum_{j=1}^{3}C_{kii}}$$

The assigned weights for different classes of different classifiers are calculated by Equation (19):

(19) $$W_{ki}=\frac{C_{ki}}{\sum_{k=1}^{2}C_{ki}}.$$

Assuming that the possible distribution of class i is $\pi_{ki}$, we then calculate the credibility of each base classifier for the class to obtain the weight of different classifiers by using Equation (20).

(20) $$\pi_{i}(\pi_{1i},\pi_{2i})=T(W_{i1}\pi_{i1},W_{i2}\pi_{i2})$$

We optimize the possibility of synthesis in confusing areas with high uncertainty. The T-module operator of q→0 is selected and normalized according to the credibility of the classifier to determine the weighted factor.

## 4. Experimental Results and Discussion

### 4.1. Feature Engineering

Considering the importance of feature extraction for classification results, this paper discussed a variety of point cloud features and their computation methods in Section 3.2. Since the selection of neighborhood radii during feature computation has a direct impact on the feature computation results, it will finally affect the classification accuracy. A different neighborhood radius may introduce different numbers of invalid points in the feature calculation, which are often contained in the original data. The number of invalid points in the feature extraction process should be kept as few as possible. A neighborhood radius of 0.5 is a more accurate representation of the actual distribution characteristics of the point cloud. By using a step size of 0.5 for the neighborhood radius, we can capture the relationships between neighboring points with greater precision, resulting in more reliable local information. Based on this, taking entropy and roughness as examples, the neighborhood sphere radius is set to 0.5, 1.0, 1.5, 2.0, and 2.5, respectively, and the number of feature invalid points under a different radius is counted as shown in Table 1 and Table 2, where R is the neighborhood radius and N is the number of invalid points. 

**Figure 4:**
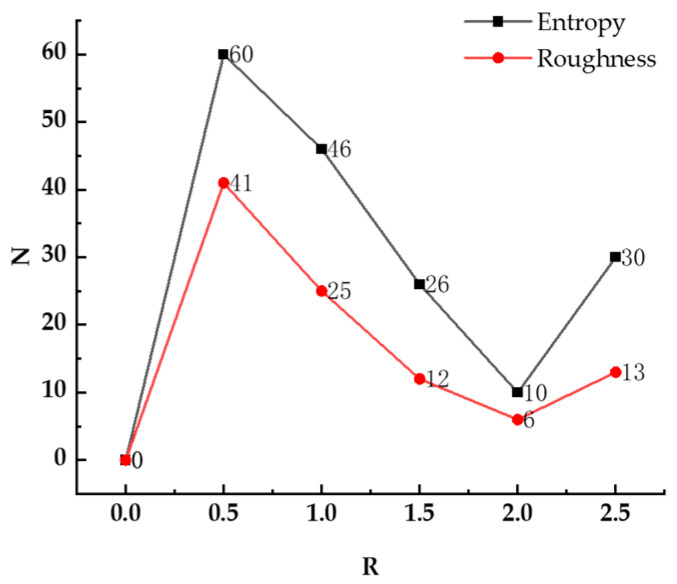
Number of invalid points.

Based on the information in Table 1 and Table 2, Figure 4 shows that when R is 2.0, the number of invalid points is the least. Therefore, in this paper, 2.0 is chosen as the best neighborhood radius to construct the feature space for SVM classification.

**Figure 5:**
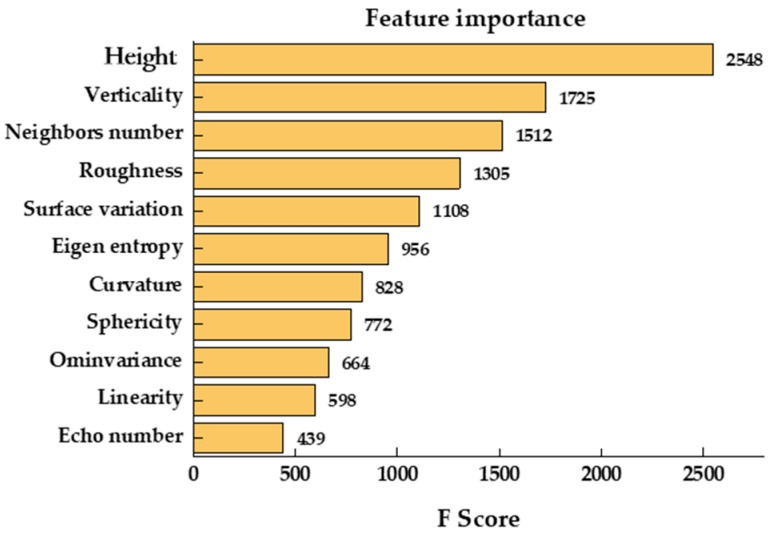
The importance of features.

The feature space contains 11 features, and the feature importance is ranked from high to low as a F score, which is given by XGBoost as shown in Figure 5. The tail-culling method is used to determine the set of features to achieve the feature dimensionality reduction that gives the highest classification accuracy; and finally, seven features are listed in text. The order of importance is shown below in Figure 5.

### 4.2. Confusing Areas

The proposed confusing areas are defined in Section 3.4 based on the range of prediction probability of base classifiers. The output probability of a base classifier is used to indicate the uncertainty to judge the degree of fuzziness of the classification to the point by the classifier. In this section, we discuss how confusing areas are determined with multiple thresholds on classification accuracy.

First, the percentage of misclassified points in our selected confusing areas was analyzed by experimentally setting [45%, 60%], [45%, 55%], [50%, 65%], and [50%, 55%] for comparison experiments. NP in Table 3 represents the sum of points in the point cloud in the given interval, FP represents the number of misclassified points in the interval, and FP/NP represents the percentage of misclassified points. 

From Table 3, it is clearly noticed that the threshold interval of [45%, 55%] covers the highest misclassification rate, but because the selected interval is small, it contains fewer misclassified points; however, when we increase the threshold interval to [45%, 60%], the number of misclassified points increases to nearly 50,000 points, with a relatively high misclassification rate.

### 4.3. Classification Results

**Figure 6:**
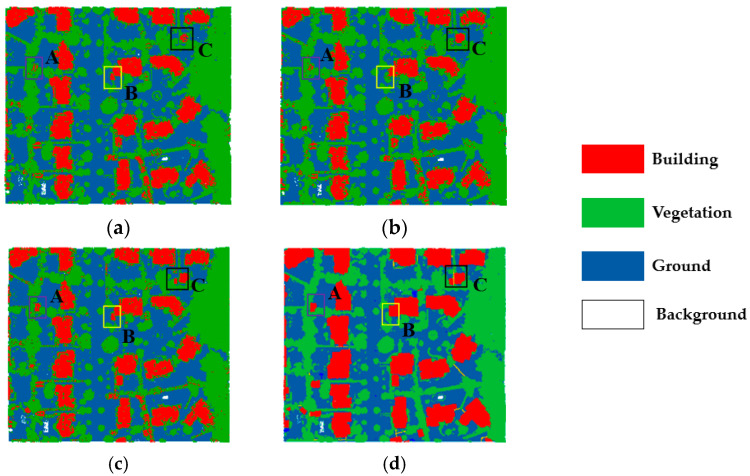
Comparison of SVM point cloud classification results of dataset 1: (**a**) SVM-RBF; (**b**) SVM-Linear; (**c**) our method; and (**d**) ground truth.

The classification results of dataset 1 are shown in Figure 6. From the subjectively visualized assessment, the classification results of our method are generally better than those from the single SVMs, and regions A, B, and C represent different confusing areas in the figure.

**Figure 7:**
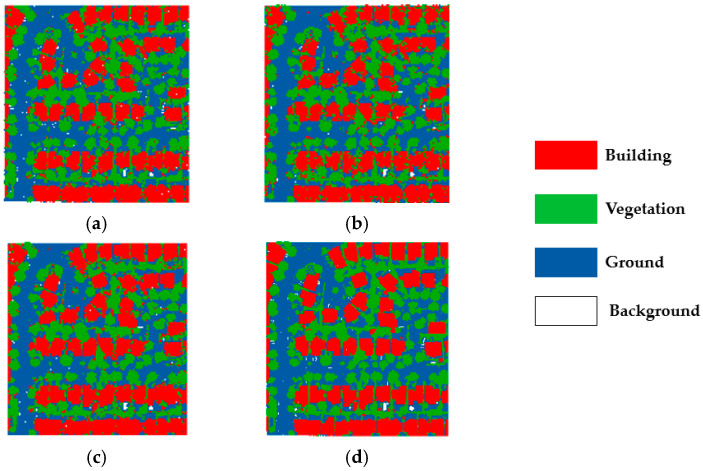
Comparison of SVM point cloud classification results of dataset 2: (**a**) SVM-RBF; (**b**) SVM- Linear; (**c**) our method; and (**d**) ground truth.

To verify the robustness of our method, we conducted the same experiment on another area in DALES. The classification results are shown in Figure 7, and it is clearly noticed that our method is closest to the manually labeled point cloud. SVM-RBF is the worst in classifying buildings, with many points being misclassified as ground.

To evaluate the classification performance quantitatively, the overall accuracy and the Kappa coefficient are used. The results of datasets 1 and 2 are shown in Table 4 and Table 5, respectively. For dataset 1, the overall accuracy of the fusion of classifiers is improved by 1.79% compared to the other methods, and Kappa coefficient is the highest, at 86.78%, among these methods. For dataset 2, the overall accuracy is improved by 1.25% and the Kappa coefficient also improved by 1.21% compared to the other methods.

**Figure 8:**
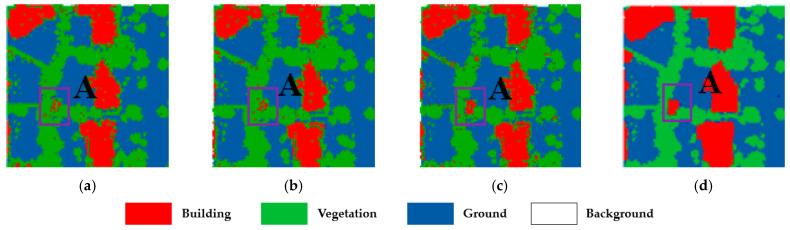
The classification results of the confusion region A: (**a**) SVM-RBF; (**b**) SVM-Linear; (**c**) our method; and (**d**) ground truth.

**Figure 9:**
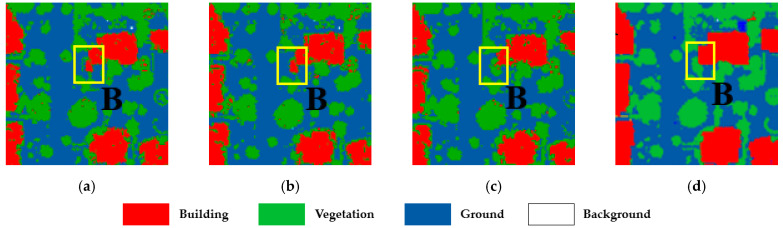
The classification results of the confusion region B: (**a**) SVM-RBF; (**b**) SVM-Linear; (**c**) our method; and (**d**) ground truth.

**Figure 10:**
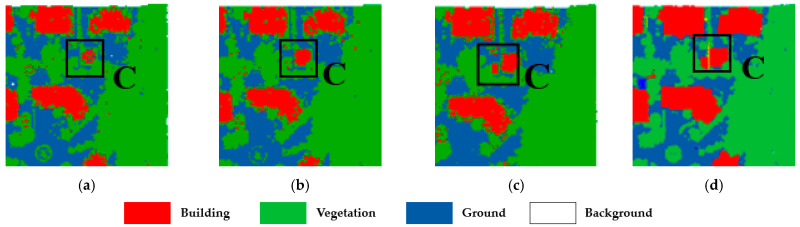
The classification results of the confusion region C: (**a**) SVM-RBF; (**b**) SVM-Linear; (**c**) our method; and (**d**) ground truth.

To validate the advantages of our method in dealing with the classification of the confusing areas, highlighted in Figure 6 labeled as A, B, and C, we enlarge the three areas and present the classification results in Figure 8, Figure 9 and Figure 10, respectively.

The three confusing areas reflect three possible scenarios in the testing area. Region A shows buildings misclassified as vegetation. This could be due to the presence of tall vegetation shading low buildings. In region B, vegetation points are largely classified as buildings. This is due to the similarity of the points of the two types of features generated from point clouds. In region C, the main problem is at the junctions between buildings, which tend to be more complex in terms of building types and tend to create areas of confusion. By fusing the classification results of different classifiers and reassigning the classes of the point cloud, the classification uncertainty is reduced. The classification accuracy is demonstrated in Table 6

The classification accuracy of the confusing areas is significantly lower than the overall classification accuracy shown in Table 4, which proves the effectiveness of the uncertainty region selection method developed in this study. The average accuracy of the proposed method in uncertain regions is improved to 69.75% from 66.40% (SVM-RBF) and 63.15% (SVM-Linear), demonstrating the effectiveness of our method in improving classification accuracy in confusing areas.

For dataset 2, the same experiment for confusing areas is conducted. With a 0.96% improvement in ground classifications over SVM-RBF and a 11.74% improvement in vegetation over SVM-Linear. The average accuracy also improves by 2.77% over the highest of the other two classifiers. The classification accuracy of the confusing areas is as shown in Table 7.

### 4.4. Discussion

As can be seen from Table 6 and Table 7, the average accuracy of the method proposed in this paper is improved in two datasets, but the ground classification accuracy is highest when the linear kernel function is used. This is because the point cloud data on the ground tend to be continuous and flat, and adjacent ground points tend to cluster at approximately the same location in feature space. This makes the ground dataset easier to delineate by linear hyperplanes in high-dimensional space. Thus, the linear kernel function has better performance in ground classification. Through techniques such as confidence-based decision making, we can combine the prediction results of multiple classifiers to obtain a more accurate final classification result. This approach improves the classification accuracy of both vegetation and buildings in our classification results by effectively reducing the bias and variance of individual classifiers.

**Figure 11:**
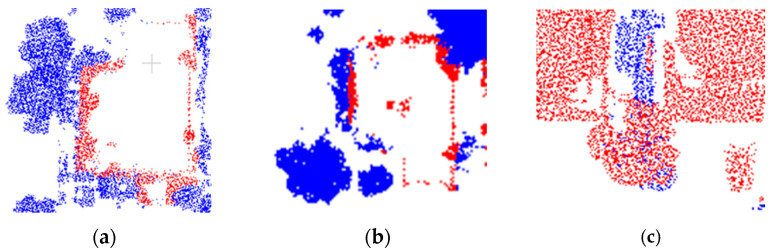
Error analysis of the confusing region of vegetation and building: (**a**–**c**) are the errors of different regions.

In Figure 6 and Figure 7, there is confusion in the overlap between the vegetation growth area and the building area, and there is a mistake in the feature calculation of the boundary points at the edges of the buildings due to the shading of the trees, resulting in the misclassification of the buildings as vegetation points. As shown in Figure 11a–c, the red areas are some building edges that were misclassified as vegetation points, and the main areas with this type of error are concentrated in the building scenes that are surrounded by dense and tall vegetation. In addition, the main reason for the omission of the vegetation area is that the buildings and tall vegetation cover the low vegetation between the buildings, which leads to the omission of the vegetation area in this part, and our method can better classify the regular building edges, but the method in this paper still has a part of the confusing that was not solved. There are still some point clouds above the trees that are misclassified as buildings because the discriminative power of the given classification features in our current feature space is not strong or even unable to discriminate between classes, which hardly meets the demand for high precision classification.

Overall, with the above error analysis and taking into account some of the limitations of the methodology in this paper, our next steps will be to continuously explore how to deal with other misclassified points in confusing areas in terms of the following measures: Firstly, the use of more effective features can be further explored, such as different point cloud feature descriptors, such as point feature histograms (PFH), fast point feature histograms (FPFH), color features, and attribute features. By introducing more features, more information can be provided to distinguish different feature classes and reduce the confusion problem. Then, the confusing area can be defined directly from the correspondence between features and classes. By analyzing the similarities and differences of features between different land cover classes, the range of feature values in the confusing area is determined. It allows features to be mapped into intervals and these intervals can be processed using uncertain information processing methods; finally, use more advanced machine learning or deep learning techniques for efficient land cover classification.

In conclusion, through the steps of exploring more effective features, identifying confusing areas, and constructing efficient classifiers, the classification model for LiDAR data can be further improved to solve the confusing problem and enhance classification accuracy.

## 5. Conclusions

In this study, we proposed a novel method for the land cover classification of LiDAR point clouds based on possibility theory and muti-classifier fusion. By optimizing the fuzzy uncertainty information in the classification process, the method integrates the advantages of different SVM classifiers and overcomes the limitations. The proposed method includes three strategies to effectively improve the classification accuracy: (1) feature space construction using the XGBoost algorithm to measure feature importance; (2) definition of the confusing area and classifier confidence based on the base classifier results; (3) weighted possibility distribution synthesis to avoid the misclassification of categories boundaries. The quantitative analysis results show that the overall accuracy of this method can reach 94.14%, the Kappa coefficient can reach 88.45%, and in confusing areas, classification accuracies of the ground, vegetation, and buildings can reach 88.20%, 73.09%, and 70.61%, respectively. Therefore, the method in this paper can improve the classification accuracy of land cover classification and can be effective in confusing areas.

However, there are still some shortcomings in this study, such as the correlation and complementarity between features, the selection of classifiers and synthesis rules, etc. As the effectiveness of deep learning models depends on the quantity and quality of data used for training, their performance is not always superior to traditional statistical methods. Therefore, we will continue to explore the application of machine learning classifiers to land cover classification in the future. The classification model of LiDAR data will be further improved by exploring more effective features, identifying the confusing area from feature analysis, and constructing an efficient classifier to increase the classification accuracy so that our method can better serve remote sensing application scenarios.

## Figures and Tables

**Table 1:** Entropy results and invalid points statistics.

Entropy	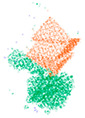	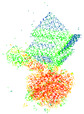	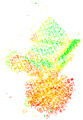	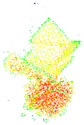	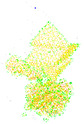	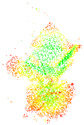
R	0	0.5	1.0	1.5	2.0	2.5
N	0	60	46	26	10	30

**Table 2:** Roughness of different radii.

Roughness	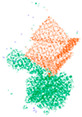	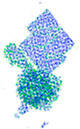	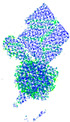	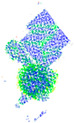	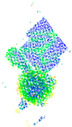	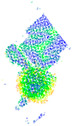
R	0	0.5	1.0	1.5	2.0	2.5
N	0	41	25	12	6	13

**Table 3:** Confusing interval comparison.

Interval	[45%, 60%]	[45%, 55%]	[50%, 65%]	[50%, 55%]
NP	92,305	54,013	115,552	32,173
FP	48,734	29,238	58,208	15,548
FP/NP	52.8%	54.13%	50.37%	48.32%

**Table 4:** Overall accuracy (%) evaluation results on test dataset 1.

		Random Forest	SVM-RBF	SVM-Linear	Our Method
Overall	Accuracy	91.35	91.37	90.25	93.16
	Kappa	85.97	86.04	85.30	86.78

**Table 5:** Overall accuracy (%) evaluation results on test dataset 2.

		Random Forest	SVM-RBF	SVM-Linear	Our Method
Overall	Accuracy	92.89	93.06	91.50	94.14
	Kappa	86.13	87.24	85.91	88.45

**Table 6:** Classification accuracy (%) of confusing areas based on test dataset 1.

Method	Ground	Vegetation	Building	Average
SVM-RBF	74.38	63.55	60.55	66.40
SVM-Linear	82.15	45.10	61.42	63.15
Our Method	77.16	67.50	64.15	69.75

**Table 7:** Classification accuracy (%) of confusing areas based on test dataset 2.

Method	Ground	Vegetation	Building	Average
SVM-RBF	87.24	70.04	66.37	74.53
SVM-Linear	89.16	61.35	69.34	73.28
Our Method	88.20	73.09	70.61	77.30

## Data Availability

Not applicable.
